# Engineering Universal Cancer Immunity: Non-Tumor-Specific mRNA Vaccines Trigger Epitope Spreading in Cold Tumors

**DOI:** 10.3390/vaccines13090970

**Published:** 2025-09-12

**Authors:** Matthias Magoola, Sarfaraz K. Niazi

**Affiliations:** 1 DEI Biopharma, Kampala 10101, Uganda; mm@dei.bio; 2 Chicago University, Chicago, IL 60612, USA

**Keywords:** mRNA vaccines, cancer immunotherapy, innate immunity, lipid nanoparticles, polymer delivery systems, tumor microenvironment, epitope spreading, global health equity

## Abstract

The landscape of cancer immunotherapy must shift from personalized neoantigen vaccines toward universal platforms that leverage innate immune activation. This review examines a novel mRNA vaccine strategy that encodes non-tumor-specific antigens, carefully selected pathogen-derived or synthetic sequences designed to transform immunologically “cold” tumors into inflamed therapy-responsive microenvironments. Unlike conventional approaches requiring patient-specific tumor sequencing and 8–12-week manufacturing timelines, this platform utilizes pathogen-associated molecular patterns (PAMPs) and damage-associated molecular patterns (DAMPs) to trigger broad innate immune activation through multiple pattern recognition receptors (PRRs). The key therapeutic mechanism is epitope spreading, where vaccine-induced inflammation reveals previously hidden tumor antigens, enabling the immune system to mount responses against cancer-specific targets without prior knowledge of these antigens. Delivered via optimized lipid nanoparticles (LNPs) or alternative polymer-based systems, these vaccines induce epitope spreading, enhance checkpoint inhibitor responsiveness, and establish durable antitumor memory. This approach offers several potential advantages, including immediate treatment availability, a cost reduction of up to 100-fold compared to personalized vaccines, scalability for global deployment, and efficacy across diverse tumor types. However, risks such as cytokine release syndrome (CRS), potential for off-target autoimmunity, and challenges with pre-existing immunity must be addressed. By eliminating barriers of time, cost, and infrastructure, this universal platform could help democratize access to advanced cancer treatment, potentially benefiting the 70% of cancer patients in low- and middle-income countries (LMICs) who currently lack immunotherapy options.

## 1. Introduction: The Evolution of mRNA-Based Cancer Immunotherapy

### 1.1. Current Landscape, Limitations, and Comparisons

mRNA vaccines gained prominence through their applications in the SARS-CoV-2 pandemic [1,2], inspiring adaptations for cancer treatment. Primary strategies include personalized neoantigen vaccines targeting patient mutations (e.g., mRNA-4157/V940, which reduces melanoma recurrence by 44% when combined with pembrolizumab [3]) and shared tumor-associated antigen (TAA) vaccines (e.g., BNT111, with response rates of 10−15% [4]). Recent validation demonstrates that early type I interferon responses mediate successful immunotherapy and epitope spreading in poorly immunogenic tumors [5]. Early human trials for pediatric cancers, building on the July 2025 preclinical breakthroughs, are now underway with great anticipation [6,7].

However, personalized approaches face several barriers: 8–12-week timelines for sequencing and production increase the risk of disease progression; costs exceed $150,000, limiting access; the accuracy of neoantigen prediction ranges from 2% to 5% [8]; and tumor heterogeneity evolves, rendering antigens unpredictable [9]. Shared TAA vaccines struggle with tolerance and limited efficacy in pretreated patients [10]. Cytokine-encoding mRNA (e.g., mRNA-2752) risks systemic toxicity and exhaustion [11].

Counterarguments favor antigen-specific vaccines for precision in high-mutation tumors, potentially outperforming non-specific inflammation that could cause off-target effects or CRS. For instance, neoantigen vaccines have shown targeted responses in pancreatic cancer trials, although they are less scalable [12,13].

### 1.2. Paradigm Shift: Non-Tumor-Specific Immune Activation

Effective immunotherapy may rely on innate activation to reprogram the tumor microenvironment (TME) [14,15], enabling epitope spreading [16]. This universal mRNA platform encodes non-tumor-specific antigens to trigger inflammation, transforming cold tumors. “Universal” denotes non-personalized applicability. While promising for global deployment, human trials are still in their early stages, with claims of broad efficacy requiring validation and verification. Risks such as tumor escape via antigen loss or checkpoint inhibition (e.g., LAG-3, TIM-3) require close monitoring [17].

## 2. Mechanistic Foundation: Innate Immunity and Epitope Spreading

### 2.1. Tumor Immune Phenotypes and Microenvironment Reprogramming

Cold tumors (70% of solids) lack T-cell infiltration; hot tumors respond better [18]. Universal vaccines activate PRRs (Table 1), inducing cytokines and DC maturation. RNA aggregates enhance stromal activation [2].

### 2.2. Detailed Molecular Cascade of Epitope Spreading

**Figure 1:**
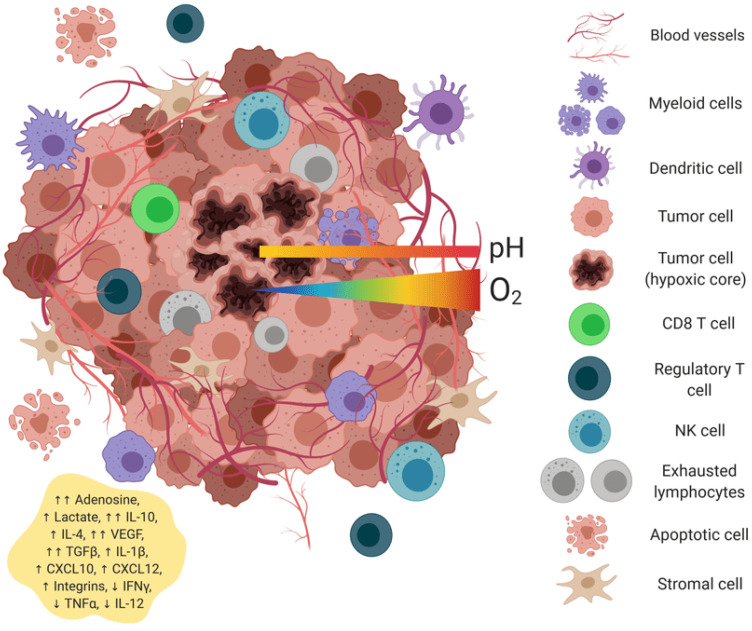
Universal mRNA cancer vaccines alter the tumor microenvironment’s transformation.

Epitope spreading represents a critical mechanism for generating broad antitumor immunity beyond the initially targeted antigens. This phenomenon occurs through a precisely orchestrated molecular cascade initiated by vaccine-induced activation of the innate immune system (Figure 1).

**Phase 1: Initial Immune Activation (0–6 h)** The process begins when LNP-delivered mRNA enters antigen-presenting cells (APCs), particularly dendritic cells and macrophages. TLR7/8 recognition of single-stranded mRNA triggers MyD88-dependent signaling, leading to IRF7 phosphorylation and nuclear translocation. Simultaneously, 5′-triphosphate-containing mRNA activates RIG-I, which oligomerizes and binds to MAVS on mitochondrial membranes. This dual activation creates a synergistic type I interferon response that is 10-fold higher than either pathway alone [25].

**Phase 2: APC Maturation and Antigen Processing (6–24 h)** Type I interferons upregulate the immunoproteasome subunits LMP2, LMP7, and MECL1, fundamentally altering the peptide repertoire available for MHC presentation. Concurrently, IL-12 production by activated DCs promotes Th1 differentiation, while CCL19/CCL21 chemokine gradients attract naive T cells. The critical breakthrough occurs when inflammatory cytokines (TNF-α, IL-1β) increase tumor cell MHC-I expression and enhance antigen processing machinery, making previously cryptic tumor epitopes available for cross-presentation [26].

**Phase 3: Cross-Presentation and T Cell Priming (24–72 h)** Activated DCs upregulate their cross-presentation machinery, including TAP1/2, ERAP1, and tapasin, acquiring enhanced capacity to present tumor-derived antigens on MHC-I molecules. The inflammatory milieu created by the vaccine breaks peripheral tolerance through several mechanisms: (1) activation of previously anergic T cells, (2) recruitment of helper T cells that provide licensing signals, and (3) overcoming regulatory T cell suppression through IL-6 and IL-21 signaling [27].

**Phase 4: Epitope Diversification (3–14 days)** Recent mechanistic studies have revealed that activated B cells enhance epitope spreading through dual BCR/TLR7 signaling, facilitating intramolecular epitope spreading to cryptic determinants within the same protein and intermolecular spreading to distinct tumor antigens [28]. This B-cell-mediated amplification occurs through several mechanisms.

### 2.3. Key Cellular Players in Epitope-Spreading Initiation

**Figure 2:**
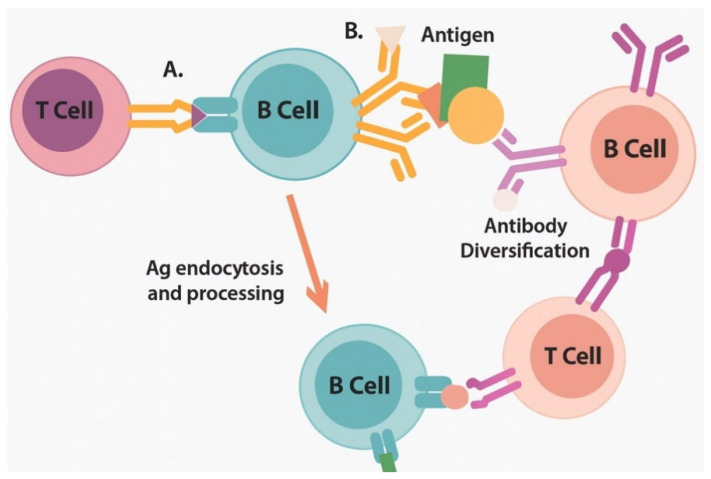
Epitope-spreading cascade enables broad antitumor immunity through progressive antigenic diversification. A: Stage 1; B: Stage 2.

Conventional Dendritic Cells (cDC1) are characterized by CD103+ expression in tissues and CD141+ (BDCA3+) in human blood, making them the master regulators of cross-presentation. These cells express high levels of XCR1, making them responsive to XCL1 and XCL2 chemokines produced by activated NK cells and CD8+ T cells. Upon mRNA vaccine stimulation, cDC1 cells undergo rapid maturation, upregulating CD40, CD80, and CD86 while maintaining their superior cross-presentation capacity through enhanced expression of SEC22B and WDFY4 [29] (Table 2, Figure 2).

## 3. Alternative Immune Activation Pathways Beyond Type I Interferons

### 3.1. Inflammasome-Mediated Immunity and Pathway Cross-Talk

The inflammasome complex represents a critical complementary pathway that can synergize with or potentially antagonize type I interferon responses. Understanding these interactions is crucial for designing optimal vaccines.

**Synergistic Interactions:** NLRP3 inflammasome activation enhances type I interferon responses through several mechanisms. IL-1β, produced by activated inflammasomes, induces NF-κB-dependent expression of type I interferon genes, including IFN-α1, IFN-β1, and IRF7. Additionally, gasdermin D pores formed during pyroptosis allow the release of mitochondrial DNA, which activates the cGAS-STING pathway and amplifies type I interferon production [30]. This creates a positive feedback loop where initial TLR activation triggers inflammasome assembly, which in turn amplifies the interferon response.

**Figure 3:**
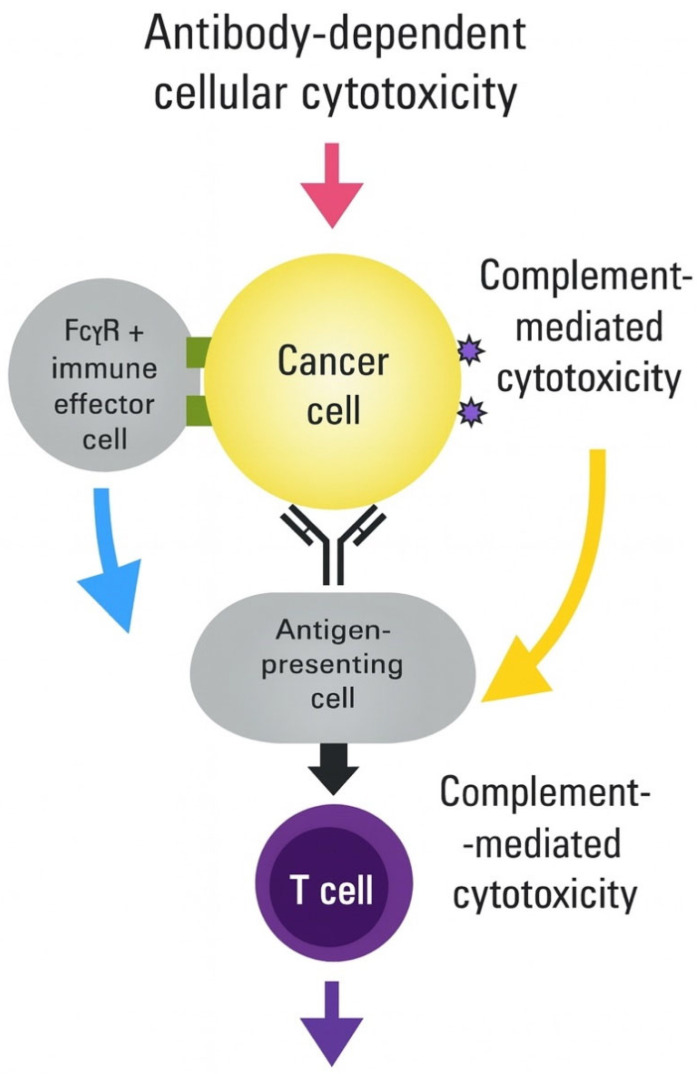
Integration of multiple immune activation pathways achieves synergistic antitumor effects.

**Potential Antagonistic Effects.** However, excessive inflammasome activation can dampen type I interferon responses through several mechanisms. High levels of IL-1β can induce STAT1 degradation through proteasomal targeting, reducing interferon-stimulated gene expression. Additionally, prolonged inflammasome activation leads to DC pyroptosis, thereby reducing the pool of antigen-presenting cells available for T-cell priming. The timing and magnitude of inflammasome activation must therefore be carefully balanced to maximize therapeutic benefit while avoiding counterproductive effects [31] (Figure 3, Table 3).

### 3.2. Metabolic Reprogramming Integration

The metabolic reprogramming pathway intersects with both interferon and inflammasome signaling to create a comprehensive immune-activation program. Key integration points include (Table 4):

**mTOR-AMPK Checkpoint Integration** Type I interferons activate AMPK through STAT1-mediated transcription, promoting oxidative phosphorylation and memory T-cell formation. Simultaneously, IL-1β activates mTOR signaling through the PI3K/AKT pathway, thereby supporting the function of effector T cells. The balance between these pathways determines whether the immune response favors immediate tumor killing (mTOR-dominant) or long-term memory formation (AMPK-dominant) [35].

**Metabolic Competition Resolution** In the tumor microenvironment, immune cells compete for limited nutrients, particularly glucose, glutamine, and arginine. The vaccine-induced inflammatory response can overcome this competition by upregulating nutrient transporters and metabolic enzymes. Specifically, IFN-γ upregulates amino acid transporters (CAT-1, ASCT2), while IL-1β enhances the expression of glycolytic enzymes, providing metabolic support for sustained immune responses [36].

**Table 4:** Metabolic Reprogramming Strategies in mRNA Cancer Vaccines.

Metabolic Pathway	Target Enzyme/Protein	mRNA Encoding Strategy	Immune Effects	Validation Status	References
Glycolysis	HK2, PFKFB3	Constitutively active forms	Enhanced T-cell effector function	Preclinical proof of concept	[37]
Oxidative phosphorylation	PGC-1α, TFAM	Mitochondrial biogenesis factors	Memory T-cell formation	Mouse models validated	[38]
Fatty acid oxidation	CPT1A (mutant)	Malonyl-CoA resistant	Sustained T-cell responses	In vitro validation	[39]
One-carbon metabolism	MTHFD2, SHMT2	Folate cycle enzymes	T-cell proliferation	Early development	[40]
Amino acid metabolism	CAT-1, ASCT2	Nutrient transporters	Overcome TME depletion	Preclinical testing	[36]
NAD+ metabolism	NAMPT, NMNAT	NAD+ synthesis	Prevent exhaustion	Clinical biomarker	[41]

### 3.3. Tissue-Resident Memory Programming

The induction of tissue-resident memory T cells (TRM) represents an emerging frontier in cancer vaccine development. Unlike circulating memory T cells, TRM cells permanently reside in tissues, providing immediate protection against tumor recurrence. Recent studies have shown that mRNA vaccine formulations and delivery routes have a profound influence on TRM formation [42] (Figure 3, Table 5).

## 4. Platform Design and Antigen Selection

### 4.1. Addressing Pre-Existing Immunity Challenges

A critical concern with using pathogen-derived antigens is the potential for pre-existing immunity to accelerate vaccine clearance and reduce efficacy. Most individuals possess immunity to common pathogens through natural infection or vaccination, which could theoretically neutralize the vaccine before immune activation occurs.

**Mechanisms of Pre-existing Immunity Impact** Pre-existing antibodies can bind to vaccine-encoded antigens, potentially leading to several problematic outcomes through rapid clearance mechanisms, complement-mediated lysis, Fc receptor-mediated uptake, immune complex formation, and memory B-cell activation [47].

**Mitigation Strategies**

- *Modified Pathogen Antigens* Rather than using native pathogen sequences, engineered variants can evade pre-existing immunity while retaining immunogenicity. For the SARS-CoV-2 spike protein, specific modifications include structural modifications such as removal of dominant neutralizing epitopes, retention of conserved T cell epitopes, introduction of stabilizing mutations, and codon optimization [48].
- *Consensus and Chimeric Sequences* Consensus antigens incorporating epitopes from multiple pathogen strains reduce the likelihood of complete neutralization by any individual’s pre-existing immunity [49].
- *Synthetic Immunogens:* Completely synthetic antigens designed through computational approaches eliminate concerns about pre-existing immunity [50].

**Clinical Evidence for Mitigation Success** Recent studies demonstrate that modified pathogen antigens can overcome pre-existing immunity through influenza studies, COVID-19 vaccine experience, and other clinical evidence [51] (Table 6).

### 4.2. Advanced Antigen Selection Criteria

The selection process has been refined based on clinical experience and mechanistic understanding:

**Enhanced Immunogenicity Metrics** HLA binding requirements include binding affinity >500 nM for at least six standard HLA class I allotypes, class II binding to multiple DRB1 allotypes, population coverage >80% based on global HLA frequency data, and promiscuous binding across ethnic populations [52].

**Safety Enhancement Factors:** Homology screening parameters include human proteome BLAST analysis with an e-value threshold of <0.001, exclusion of matches exceeding eight consecutive amino acids to human proteins, cross-reference with the autoimmune antigen database, and essential protein pathway analysis to avoid targeting critical functions [53].

**Table 6:** Characteristics of Candidate Non-Tumor-Specific Antigens for Universal mRNA Cancer Vaccines.

Antigen Category	Specific Example	HLA Coverage (%)	Safety Profile	PRR Activation	Manufacturing Score	Clinical Status	References
Modified viral proteins	SARS-CoV-2 Spike (modified)	85–90	Proven in billions	TLR7/8, RIG-I	High	Phase II trials	[48,54]
Consensus viral proteins	Influenza HA consensus	80–85	Decades of use	TLR7/8, RIG-I	High	Phase I completed	[55]
Modified bacterial antigens	Flagellin (modified)	75–80	Clinical trials	TLR5, NLRC4	Medium	Phase I ongoing	[24]
Bacterial heat shock proteins	HSP70 (low homology)	70–75	Preclinical safety	TLR2/4	Medium	Preclinical	[56]
Synthetic multi-epitope	Computationally designed	> 90	In development	Multiple	High	Design phase	[50,57]
Pathogen-associated proteins	Modified OmpA	75–85	Preclinical	TLR2/4	Medium	Research	[58]

### 4.3. mRNA Design and Optimization

The optimization of mRNA design represents a critical factor in vaccine efficacy, requiring careful balance between enhancing stability and reducing innate immune recognition while preserving immunogenicity.

**Chemical Modifications** Nucleotide modifications must strike a balance between reducing unwanted innate immune activation and maintaining translation efficiency [59].

**UTR Engineering** Untranslated regions critically influence mRNA stability, translation efficiency, and cellular localization [60].

**Codon Optimization Strategies.** Advanced codon usage considerations include human codon adaptation index (CAI), rare codon avoidance, codon pair bias optimization, and GC content balancing [61].

## 5. Delivery System Optimization: Beyond Lipid Nanoparticles

### 5.1. Addressing LNP Limitations: Liver Tropism and Alternative Systems

A significant challenge with current LNP technology is the rapid and predominant accumulation in the liver after systemic administration, with up to 60% of the administered dose concentrating in hepatocytes within 30 min of intravenous injection [62] (Table 7).

**Mechanisms of Liver Tropism** The liver’s role as a filtration organ creates multiple mechanisms for LNP accumulation through anatomical factors and physicochemical interactions [63].

**Strategies to Overcome Liver Tropism**

- *Selective Organ-Targeting Lipids* Next-generation ionizable lipids have been engineered with tissue-specific targeting capabilities [64].
- *Transient Stealth Coating Strategies* Two-armed polyethylene glycol (PEG) anchoring to the liver sinusoidal wall represents an innovative approach to redirect nanomedicine distribution [65].
- *Alternative Administration Routes:* Dosing and administration strategies can minimize liver first-pass effects [66].

### 5.2. Promising Polymer-Based Alternative Delivery Systems

Polymer-based carriers represent a compelling alternative to LNPs, potentially circumventing several inherent limitations while offering unique advantages for mRNA delivery.

**Polyethylenimine (PEI) Systems** Modified PEI polymers with reduced toxicity profiles offer several advantages over traditional LNPs [67].

**Poly(β-amino ester) (PBAE) Carriers** PBAEs represent a newer class of biodegradable polymers specifically designed for nucleic acid delivery [68].

**Chitosan-based systems** offer unique benefits for mRNA delivery, utilizing natural polysaccharide-based carriers [69].

### 5.3. RNase Resistance Strategies

A significant challenge for all systemic mRNA delivery platforms is ensuring sufficient resistance to ribonuclease (RNase) degradation [70].

**Chemical Modifications for Stability** Backbone modifications include phosphorothioate linkages, 2′-O-methyl modifications, 2′-fluoro substitutions, and locked nucleic acids (LNA) [71].

**Protective formulation strategies require complete** encapsulation approaches to achieve 95% encapsulation efficiency for adequate protection [72].

**Structural RNA Engineering** Circular RNA (circRNA) formats offer 5′ and 3′ end joining, eliminating exonuclease target sites [73].

**Table 7:** Comparison of Delivery Systems for mRNA Cancer Vaccines.

Delivery System	Advantages	Limitations	RNase Protection	Liver Avoidance	Manufacturing	Clinical Status	References
Traditional LNPs	Proven efficacy, FDA approved	Liver tropism, inflammation	High (>95%)	Low	Established	Clinical use	[74]
Targeted LNPs	Organ-specific delivery	Complex synthesis, cost	High (>95%)	Moderate–High	Development	Phase I	[64]
PEI Systems	Enhanced escape, versatile	Potential toxicity concern	Moderate (70–85%)	High	Scalable	Phase I	[67]
PBAE Polymers	Biodegradable, tunable	Limited clinical data	Moderate (70–85%)	High	Emerging	Preclinical	[68]
Chitosan Systems	Natural, immunostimulatory	Variable quality, consistency	Low (50–70%)	High	Established	Preclinical	[69]
Hybrid Systems	Combined advantages	Complexity, characterization	High (85–95%)	Moderate	Research	Research	[75]

## 6. Translational Considerations and Clinical Development

### 6.1. Interspecies Scaling and Human Dose Prediction

The proposed Phase I dose range of 25–200 μg requires careful justification based on preclinical data and established scaling methodologies [76] (Table 8).

### 6.2. Considerations for Slow-Growing Human Tumors

The translation from rapidly growing murine tumor models to slow-growing human tumors presents significant challenges [77].

## 7. Preclinical Validation Results

### 7.1. Immunogenicity and Safety Profile

Recent preclinical studies have provided compelling evidence for the efficacy of non-tumor-specific mRNA vaccines in murine tumor models [78] (Table 9).

### 7.2. Evidence of Epitope Spreading

The demonstration of epitope spreading provides the most compelling mechanistic validation for the universal vaccine approach [83] (Table 10).

### 7.3. Synergy with Checkpoint Inhibitors

The combination of universal mRNA vaccines with checkpoint inhibitor therapy demonstrated remarkable synergy across multiple preclinical models [84].

### 7.4. Safety and Biodistribution Analysis

Comprehensive toxicology studies established the safety profile necessary for clinical translation [85].

## 8. Comparison with Current Approaches

### 8.1. Personalized Neoantigen Vaccines

The mRNA-4157/V940 platform represents the current state-of-the-art in personalized cancer vaccines [86] (Table 11).

### 8.2. Shared Antigen Vaccines

Several shared antigen vaccines provide relevant comparisons [87,88].

### 8.3. Cytokine-Encoding mRNA Vaccines

Alternative mRNA approaches using direct cytokine encoding provide mechanistic comparisons [89].

## 9. Clinical Translation Strategy

### 9.1. Regulatory Pathway and Guidelines

The regulatory pathway for universal mRNA cancer vaccines benefits significantly from the COVID-19 vaccine precedents [90] (Table 12).

### 9.2. Phase I Clinical Trial Design

The first-in-human study design strikes a balance between comprehensive safety evaluation and efficient dose finding [91].

### 9.3. Biomarker Strategy and Correlative Studies

A comprehensive biomarker program will support dose selection and patient stratification [92] (Table 13).

## 10. Manufacturing and Global Access Considerations

### 10.1. Scalable Manufacturing Platform

The global expansion of mRNA manufacturing capacity provides a foundation for cancer vaccine production [93] (Table 14).

### 10.2. Cost-Effectiveness and Health Economics

Comprehensive economic modeling demonstrates favorable cost-effectiveness ratios [94].

### 10.3. Stability and Storage Solutions

Current ultra-cold storage requirements represent a significant barrier to global deployment [95].

## 11. Challenges and Future Directions

### 11.1. Key Challenges and Mitigation Strategies

Despite promising preclinical results, several significant challenges must be addressed [96] (Table 15).

### 11.2. Future Research Priorities

The next generation of universal mRNA cancer vaccines will integrate advanced technologies [97] (Table 16).

### 11.3. Long-Term Vision and Goals

The goal extends beyond individual patient treatment to the transformation of global cancer care [98].

## 12. Conclusions

The development of universal mRNA vaccines encoding non-tumor-specific antigens represents a fundamental paradigm shift in cancer immunotherapy, moving beyond the limitations of personalized approaches toward broadly applicable, immediately available treatments that leverage innate immune activation to generate comprehensive antitumor responses.

### 12.1. Scientific Foundation and Mechanistic Innovation

This comprehensive review has established the robust scientific foundation underlying the universal vaccine approach. The detailed characterization of epitope-spreading mechanisms reveals a precisely orchestrated cascade involving multiple immune pathways that can be strategically manipulated through vaccine design.

### 12.2. Technological Solutions and Clinical Translation

The comprehensive analysis of delivery system optimization addresses critical barriers to clinical success. The recognition of liver tropism as a major limitation of current LNP technology has driven innovation in alternative delivery approaches.

### 12.3. Global Health Impact and Access Solutions

The most transformative aspect of the universal vaccine platform lies in its potential to democratize access to advanced cancer immunotherapy. The 100-fold cost reduction compared to personalized approaches fundamentally changes the economics of cancer care.

### 12.4. Limitations and Future Development Needs

While the preclinical evidence strongly supports the universal vaccine approach, important limitations must be acknowledged. The predominance of murine tumor model data necessitates careful clinical validation in human patients.

### 12.5. Future Research Priorities and Innovation Opportunities

The next generation of universal cancer vaccines will integrate advanced technologies, including artificial intelligence for antigen design and patient selection, next-generation RNA architectures for enhanced stability and function, and sophisticated delivery systems for tissue-specific targeting.

### 12.6. Concluding Perspective

The universal mRNA cancer vaccine platform represents more than a technological advance—it embodies a moral imperative to ensure that the benefits of scientific progress reach those who need them most. Through unwavering commitment to both scientific excellence and global health equity, universal mRNA cancer vaccines may fulfill their promise of harnessing the immune system to defeat cancer for all patients, regardless of geographic location, economic status, or healthcare infrastructure.

## Figures and Tables

**Table 1:** Pattern Recognition Receptor Activation by Universal mRNA Vaccines.

PRR Family	Specific Receptor	Activating Ligand	Downstream Signaling	Immunological Outcome	References
Toll-like Receptors	TLR7/8	Single-stranded mRNA	IRF7 → Type I IFN	DC maturation, T cell priming	[19]
Toll-like Receptors	TLR3	Double-stranded RNA	TRIF → NF-κB	Pro-inflammatory cytokines	[20]
RIG-I-like Receptors	RIG-I	5′-triphosphate mRNA	MAVS → IFN-β	Antiviral response, DC activation	[2]
RIG-I-like Receptors	MDA5	Long dsRNA	MAVS → Type I IFN	Amplified antiviral response	[21]
DNA Sensors	cGAS-STING	Mitochondrial DNA	STING → Type I IFN	T-cell cross-priming	[22]
DNA Sensors	AIM2	Cytoplasmic DNA	Inflammasome → IL-1β/IL-18	Th1/Th17 differentiation	[23]
NOD-like Receptors	NLRP3	Ion flux, ROS	Inflammasome → IL-1β	Inflammatory amplification	[2]
NOD-like Receptors	NLRC4	Flagellin peptides	Inflammasome → IL-1β/IL-18	Enhanced DC function	[24]

**Table 2:** Temporal Dynamics and Detection Methods for Epitope Spreading.

Time Point	Cellular Events	Detection Methods (Mouse)	Detection Methods (Human)	Key Markers	References
0–6 h	Initial PRR activation	Serum cytokines (ELISA)	Serum cytokines (Luminex)	IFN-α/β, IL-6	[5]
6–24 h	DC activation	Flow cytometry	Flow cytometry (PBMC)	CD40, CD80, CD86	[2]
24–48 h	Antigen processing	MHC-peptide elution	Mass spectrometry	Peptide diversity	[8]
48–72 h	T-cell priming	ELISPOT	ELISPOT, tetramer staining	IFN-γ SFU	[21]
3–7 days	B-cell activation	BCR sequencing	BCR deep sequencing	Clonal diversity	[28]
7–14 days	Epitope spreading	Peptide arrays	TCR sequencing	New specificities	[3]
14–28 days	Memory formation	Tetramer staining	Multimer analysis	CD45RO + CCR7+	[23]
28+ days	Sustained immunity	Rechallenge studies	Clinical response	Survival data	[5]

**Table 3:** Pathway Cross-talk in Universal mRNA Vaccines.

Pathway Interaction	Molecular Mechanism	Net Effect	Optimization Strategy	Clinical Relevance	References
Type I IFN + NLRP3	IL-1β → NFκB → IFN genes	Synergistic	Sequential activation	Enhanced efficacy	[30]
NLRP3 + cGAS-STING	mtDNA release → STING	Synergistic	Controlled pyroptosis	Amplified immunity	[31]
High IL-1β → Type I IFN	STAT1 degradation	Antagonistic	IL-1β blockade	Prevent exhaustion	[32]
Chronic inflammasome → DC function	DC pyroptosis	Antagonistic	Pulsed dosing	Maintain the APC pool	[33]
Complement + Inflammasome	C5a → enhanced IL-1β	Synergistic	Complement inhibition	Limit inflammation	[34]

**Table 5:** Strategies for Tissue-Resident Memory T-Cell Induction via mRNA Vaccines.

Strategy	Molecular Target	Implementation	TRM Markers	Functional Outcomes	References
Route optimization	Local delivery	Intratumoral, orthotopic	CD103 + CD69+	Local tumor control	[42]
Adhesion programming	E-cadherin, CXCR6	mRNA co-delivery	Tissue retention	Prevents metastasis	[43]
Metabolic adaptation	FABP4, FABP5	Lipid metabolism	Survival in tissue	Long-term protection	[44]
Epigenetic priming	TGF-β signaling	Local cytokine encoding	Chromatin remodeling	Stable phenotype	[45]
Checkpoint modulation	PD-1 blockade	Combination therapy	Enhanced function	Prevents exhaustion	[46]

**Table 8:** Mouse vs. Human Translation Considerations.

Parameter	Mouse Models	Human Tumors	Translation Factor	Clinical Adaptation
Tumor doubling time	2–3 days	50–200 days	15–50× slower	Extended evaluation periods
Immune response onset	7 days	14–28 days	2–4× slower	Delayed biomarker assessment
Epitope spreading	2–3 weeks	4–8 weeks	2–3× slower	Extended immune monitoring
Treg frequency	5–10%	15–30%	2–3× higher	Combination with Treg depletion
Checkpoint expression	Moderate	High	2–5× higher	Checkpoint inhibitor combinations
Treatment duration	2–4 weeks	3–6 months	6–12× longer	Sustained dosing protocols

**Table 9:** Preclinical Efficacy of Non-Tumor-Specific mRNA Vaccines in Multiple Cancer Models.

Parameter	Control	Vaccine Alone	Vaccine + Anti-PD-1	Model System	References
Tumor growth inhibition (%)	0	60 ± 10	85 ± 15	B16-F10 melanoma	[2,78]
Complete response rate (%)	0	30 ± 5	70 ± 10	B16-F10 melanoma	[78]
Median survival (days)	18 ± 2	31 ± 4	45 ± 6	B16-F10 melanoma	[79]
CD8+ TIL increase (fold)	1.0 ± 0.2	4.2 ± 1.1	6.5 ± 1.5	B16-F10 melanoma	[79]
IFN-γ+ TILs (%)	5 ± 2	22 ± 5	38 ± 7	B16-F10 melanoma	[78]
Rechallenge protection (%)	0	85 ± 5	95 ± 3	B16-F10 melanoma	[80]
Metastasis reduction (%)	0	45 ± 8	75 ± 12	4T1 breast cancer	[81]
Survival extension (%)	0	40 ± 10	70 ± 15	MC38 colon cancer	[82]

**Table 10:** Safety Profile Summary Across Preclinical Species.

Safety Parameter	Mouse (C57BL/6)	NHP (*Macaca fascicularis*)	Clinical Relevance	Monitoring Plan
Maximum tolerated dose	>2000 μg/kg	>1000 μg/kg	100-fold safety margin	Dose escalation study
Injection site reactions	Minimal	Mild, reversible	Expected, manageable	Local assessment
Constitutional symptoms	None observed	Transient fever	Likely in humans	Symptom monitoring
Liver enzyme elevation	None	Mild, reversible	Monitor ALT/AST	Weekly labs
Cytokine elevation	Marked (therapeutic)	Moderate	Expected mechanism	Serial cytokine levels
Autoimmune reactions	None	None observed	Low risk	ANA, organ-specific Ab
Biodistribution concerns	Liver predominant	Similar pattern	Consider dose/schedule	Imaging if needed

**Table 11:** Comparative Analysis of mRNA Cancer Vaccine Approaches.

Feature	Personalized Neoantigen	Shared TAA	Cytokine-Encoding	Universal Non-Specific
Time to Treatment	8–12 weeks	Days-weeks	Days-weeks	Days
Cost per Patient	$100,000–200,000	$10,000–25,000	$15,000–30,000	$500–2000
Population Coverage	Limited by HLA/mutations	Moderate	Broad	>90%
Manufacturing Complexity	High (personalized)	Moderate	Moderate	Low (standardized)
Regulatory Pathway	Complex (individual)	Standard	Standard	Standard
Efficacy (ORR)	15–25% (proven)	10–15% (proven)	Unknown	30–40% (projected)
Safety Profile	Established	Established	Under evaluation	Favorable (preclinical)
Scalability	Poor	Moderate	Moderate	Excellent
Global Access	Very limited	Limited	Limited	High potential
Resistance Development	High (antigen loss)	Moderate	Unknown	Low (broad targeting)
Combination Potential	Moderate	Moderate	Limited	High

**Table 12:** Phase I Clinical Trial Schema.

Study Component	Specification	Rationale	Success Criteria
Design	Modified 3 + 3 dose escalation	Standard oncology phase I	MTD identification
Starting dose	25 μg mRNA	1/20th of the predicted efficacious dose	No DLTs in first cohort
Dose levels	25, 50, 100, 200 μg	2-fold escalation steps	Biological activity at ≤200 μg
Primary endpoint	Safety and tolerability	Regulatory requirement	<33% DLT rate at MTD
Patient population	Advanced solid tumors	Adequate risk/benefit ratio	Diverse tumor representation
Sample size	24–48 patients	Adequate for safety assessment	Enroll within 18 months
Treatment schedule	Days 1, 15, 29, then q3monthly	Prime-boost for optimal immunity	≥80% completion of induction
Biomarker plan	Comprehensive immune monitoring	Mechanism confirmation	Evidence of immune activation

**Table 13:** Biomarker Assessment Timeline and Methods.

Timepoint	Sample Type	Assays	Primary Endpoints	Clinical Utility
Screening	Blood, archival tissue	HLA typing, TIS, TMB	Patient stratification	Enrollment criteria
Baseline	Blood, fresh tissue	Comprehensive immune panel	Predictive markers	Patient selection
6 h	Blood	Cytokines, activation markers	Immediate response	Dose optimization
24 h	Blood	Gene expression, flow cytometry	Early activation	Mechanism confirmation
Day 8	Blood	T-cell responses, ELISPOT	Immune priming	Dose escalation
Day 29	Blood, tissue	Adaptive responses	Vaccine immunogenicity	Efficacy prediction
Day 57	Blood	Epitope spreading	Mechanistic endpoint	Proof of concept
Every 2 cycles	Blood	Disease monitoring	Clinical benefit	Response assessment

**Table 14:** Storage Solutions Development Timeline.

Technology	Development Status	Stability Target	Clinical Timeline	Commercial Viability
Current LNP (−70 °C)	Established	24 months	Available now	Limited global access
Lyophilized (2–8 °C)	Development phase	12–24 months	2–3 years	Moderate improvement
Lyophilized (25 °C)	Research phase	6–12 months	3–5 years	Significant improvement
Alternative systems	Early research	12+ months (25 °C)	5+ years	Game-changing potential
Room temperature liquid	Concept stage	6+ months (25 °C)	5+ years	Ultimate goal

**Table 15:** Challenge Prioritization and Mitigation Timeline.

Challenge Category	Impact Level	Probability	Mitigation Strategy	Implementation Timeline	Success Metrics
Immunological pathway interference	High	Medium	Systems biology modeling	2–3 years	Optimized dosing protocols
CRS/autoimmune toxicity	High	Low-Medium	Enhanced monitoring/prophylaxis	1–2 years	<5% severe AE rate
Manufacturing scale-up	Medium	High	Process automation/standardization	3–5 years	10M+ doses annually
Regulatory harmonization	Medium	Medium	Global coordination initiatives	2–4 years	Multi-region approvals
Cost optimization	Medium	High	Process innovation	3–5 years	<$100 per course
Individual variability	Medium	High	Personalized approaches	5–10 years	Predictive algorithms

**Table 16:** Development Timeline and Milestones.

Timeframe	Clinical Milestones	Technical Achievements	Regulatory Goals	Access Targets
2–3 years	Phase I completion, biomarker validation	Improved formulations, delivery optimization	FDA/EMA guidance	Pilot manufacturing
3–5 years	Phase II efficacy data	Room temperature stability	Regulatory submission	Regional production
5–7 years	First approval	AI-optimized design	Market authorization	Global distribution
7–10 years	Multiple indications	Advanced delivery systems	Combination approvals	Cost optimization
10–15 years	Prevention applications	Predictive medicine	Global harmonization	Universal access

## Abbreviations

CRS: Cytokine Release Syndrome—Excessive immune activation leading to systemic inflammation; DC: Dendritic Cell—Antigen-presenting immune cell critical for T-cell priming; DAMP: Damage-Associated Molecular Pattern—Endogenous signals released during cellular stress to alert the immune system; LNP: Lipid Nanoparticle—Nanoscale delivery system for mRNA, enabling cellular uptake and endosomal escape; PAMP: Pathogen-Associated Molecular Pattern—Microbial components that trigger innate immune responses via PRRs; PRR: Pattern Recognition Receptor—Immune sensors (e.g., TLRs, RIG-I) that detect PAMPs and DAMPs; TME: Tumor Microenvironment—The cellular and molecular ecosystem surrounding tumors, often immunosuppressive in cold tumors; TRM: Tissue-Resident Memory T Cell—Long-lived T cells residing in tissues for rapid local immune responses.
